# Some Therapeutic Indications of Codeine

**Published:** 1894-06

**Authors:** 


SOME THERAPEUTIC INDICATIONS OF CODEINE.—Dr. J. Braithwaite (*Med. Week.*, 1894; II.; 132.) This clinician, who is obstetric physician and surgeon to the Leeds General Infirmary, reports that he has found by experience that codeine exerts a very favorable action on nocturnal paroxysms of laryngeal cough, on vomiting of whatever origin, and lastly on a form of diarrhea peculiar to old women, and which is characterized by the sudden occurrence on getting out of bed in the morning of a violent desire to evacuate the bowels, followed by one or two liquid stools.

For nocturnal spasms of laryngeal cough, Dr. B. administers, two hours before going to bed, four to six centigrammes (two-thirds to one grain) of codeine. This treatment insures to the patient a perfectly quiet night, untroubled by fits of coughing, without unpleasant after-effects.

Vomiting is quickly stopped, it is stated, by means of a dose of fifteen milligrammes to three centigrammes (one-fourth to one-half grain) of codeine, repeated in the former quantity four times, and in the latter twice daily, and administered in an effervescent mixture of some kind.

As regards the diarrhea of elderly women, it may be relieved in a short time by taking at about four o'clock in the morning a pill containing from three to four centigrammes (one-half to two-thirds of a grain) of codeine.

If a solution of codeine is preferred, a small quantity only is prepared at a time, dissolving it with the aid of heat, and adding a little spirit. In cold weather it should be kept in a room with a fire.—*Ex.*

